# Investigating the Vascular Phenotype of Subcutaneously and Orthotopically Propagated PC3 Prostate Cancer Xenografts Using Combined Carbogen Ultrasmall Superparamagnetic Iron Oxide MRI

**DOI:** 10.1097/RMR.0000000000000102

**Published:** 2016-10-05

**Authors:** Jake S. Burrell, Simon Walker-Samuel, Jessica K.R. Boult, Lauren C.J. Baker, Yann Jamin, Jane Halliday, John C. Waterton, Simon P. Robinson

**Affiliations:** ∗ Cancer Research UK Cancer Imaging Centre, Division of Radiotherapy and Imaging, The Institute of Cancer Research, Surrey; † Centre for Advanced Biomedical Imaging, Department of Medicine and Institute of Child Health, University College London, London; ‡ R&D Personalised Healthcare & Biomarkers, AstraZeneca, Alderley Park, Macclesfield, UK.

**Keywords:** drug delivery, tumor, ultrasmall superparamagnetic iron oxide, vasculature

## Abstract

The aim of this study was to use the combined carbogen-ultrasmall superparamagnetic iron oxide (CUSPIO) magnetic resonance imaging (MRI) method, which uses spatial correlations in independent susceptibility imaging biomarkers, to investigate and compare the impact of tumor size and anatomical site on vascular structure and function *in vivo*. Mice bearing either subcutaneous or orthotopic PC3 LN3 prostate tumors were imaged at 7 T, using a multi-gradient echo sequence to quantify R_2_^∗^, before and during carbogen (95% O_2_/5% CO_2_) breathing, and subsequently following intravenous administration of USPIO particles. Carbogen and USPIO-induced changes in R_2_^∗^ were used to inform on hemodynamic vasculature and fractional blood volume (%), respectively. The CUSPIO imaging data were also segmented to identify and assess five categories of R_2_^∗^ response. Small and large subcutaneous and orthotopic tumor cohorts all exhibited significantly (*P* < 0.05) different median baseline R_2_^∗^, ΔR_2_^∗^_carbogen_, and fractional blood volume. CUSPIO imaging showed that small subcutaneous tumors predominantly exhibited a negative ΔR_2_^∗^_carbogen_ followed by a positive ΔR_2_^∗^_USPIO_, consistent with a well perfused tumor vasculature. Large subcutaneous tumors exhibited a small positive ΔR_2_^∗^_carbogen_ and relatively low fractional blood volume, suggesting less functional vasculature. Orthotopic tumors revealed a large, positive ΔR_2_^∗^_carbogen_, consistent with vascular steal, and which may indicate that vascular function is more dependent on site of implantation than tumor size. Regions exhibiting significant ΔR_2_^∗^_carbogen_, but no significant ΔR_2_^∗^_USPIO_, suggesting transient vascular shutdown over the experimental timecourse, were apparent in all 3 cohorts. CUSPIO imaging can inform on efficient drug delivery via functional vasculature *in vivo*, and on appropriate tumor model selection for pre-clinical therapy trials

The combined carbogen ultrasmall superparamagnetic iron oxide (USPIO) imaging protocol (CUSPIO) combines two magnetic resonance imaging (MRI) techniques, intrinsic susceptibility contrast MRI and contrast-enhanced susceptibility MRI with USPIO particles. The transverse relaxation rate (R_2_^∗^) slows with high oxygen content gas breathing, which decreases the ratio of deoxyhemoglobin (paramagnetic) to oxyhemoglobin (diamagnetic) in blood. The resulting change in R_2_^∗^, ΔR_2_^∗^_carbogen_, reflects changes in oxygenation and the hemodynamic response in functional vasculature. Intravenous injection of USPIO particles increases R_2_^∗^ in tissue surrounding blood vessels that are perfused with blood plasma at the time of injection. With a known blood concentration of USPIO particles, ΔR_2_^∗^_USPIO_ can be used to calculate fractional blood volume. Given the difference in size of erythrocytes and USPIO particles, (∼6 and 0.03 μm, respectively),^1^ the volume of distribution influenced by hyperoxia and USPIO is likely to differ, with small vessels offering preferential access to USPIO particles. By combining, into a single imaging session, sequential measurements of ΔR_2_^∗^ induced by carbogen breathing and USPIO particles, greater information about vascular function and structure can be deduced than could be achieved using each technique individually.^2^ The CUSPIO protocol achieves this by implementing a novel segmentation scheme, in which ΔR_2_^∗^ estimates are compared and assigned to 1 of 5 response categories. Our previous work has shown that these categories provide distinct information about vascular function, the spatial distribution of plasma perfusion and transient hypoxia, and that these biomarkers can inform on tumor response to anti-angiogenic therapy.^2,3^

The clinical relevance of rodent models in both fundamental cancer research and drug development has been an ongoing debate for many years. The inadequacies and limitations of ectopic (subcutaneous) models are frequently highlighted, but there has also been a historical reluctance to adopt more complex models in cancer research.^4–6^ Today, more sophisticated orthotopically propagated tumor models, in which cancer cells are implanted and grown within the organ from which they were derived, and genetically engineered mouse (GEM) models, in which tumors are driven by the expression of the target of interest and arise spontaneously within the native tissue of origin, are being increasingly exploited.^7,8^ These models more faithfully emulate human tumor growth, tumor-host stromal interactions and vasculature, metastatic potential, and therapeutic response *in vivo*. The systematic use of orthotopic and GEM models in pre-clinical cancer research demands noninvasive anatomical and functional imaging methods for the longitudinal monitoring of tumor progression and response, which can also accurately inform on key hallmarks of cancer, such as angiogenesis. We have previously demonstrated the use of several MRI strategies with which to assess, and reveal differences, in the vascular phenotype and response of ectopic, orthotopic, and GEM models.^9–15^

The purpose of this study was to exploit CUSPIO imaging to investigate vascular function and structure in small and large subcutaneous, and orthotopically propagated PC3 LN3 prostate xenografts, and compare the differences in these characteristics between the different models.

## METHODS

### Cell Culture

Human PC3 LN3 prostate tumor cells, originally generated in-house from lymph node metastases arising in nude mice bearing orthotopic PC3 tumors,^16^ were cultured in T75 cm^3^ flasks (Corning Life Sciences, High Wycombe, UK) in Dubbecco's Modified Eagles Medium (DMEM; Invitrogen, Paisley, UK) supplemented with 10% (v/v) fetal bovine serum, 5 mM L-glutamine,100 IU/mL penicillin, and 100 μg/mL streptomycin (all Invitrogen). Cells were maintained at 37°C in a humidified 5% CO_2_ atmosphere.

### Tumor Propagation

All experiments were performed in accordance with the local ethical review panel, the UK Home Office Animals (Scientific Procedures) Act 1986, the United Kingdom National Cancer Research Institute guidelines for animal welfare in cancer research,^17^ and the Animal Research: Reporting of *In Vivo* Experiments (ARRIVE) guidelines.^18^ Male NCr nude mice under isoflurane anesthesia were injected with 1 × 10^6^ PC3 LN3 cells subcutaneously on the right flank, or 1 × 10^5^ cells into the ventral prostate gland.^9^ The subcutaneously inoculated animals were randomized into 2 groups of 6 mice, to be imaged as small and large tumors. Animals from group 1 were imaged 1 week later, and animals from group 2 were imaged 3 weeks later. Orthotopically inoculated animals were monitored by palpation and imaged after 3 weeks (n = 5). No adverse effects were observed in any of the mice, and none excluded from the study.

### Combined Carbogen-Ultrasmall Superparamagnetic Iron Oxide Imaging Protocol

Tumor-bearing mice were anesthetised by an intraperitoneal injection of 10 mL/kg of fentanyl citrate (0.315 mg/mL) along with fluanisone (10 mg/ml) (Hypnorm; Janssen Pharmaceutical, High Wycombe, UK), midazolam (5 mg/mL) (Hypnovel; Roche, Burgess Hill, UK), and sterile water (1 : 1 : 2). A lateral tail vein was cannulated with a 27G butterfly catheter (Venisystems; Abbot Laboratories, Maidenhead, UK) for remote administration of USPIO particles. A nosepiece was positioned for delivery of air or carbogen (95% O_2_/5% CO_2_) at a flow rate of 1 L/min. During MRI, all mice were restrained using dental paste in order to limit respiratory motion artefacts.^19^ A warm air blower was used to maintain the animal's core temperature at 37°C within the magnet bore.

MRI was performed on a 7-T horizontal bore microimaging system (Bruker, Ettlingen, Germany) using a 3 cm birdcage coil. T_2_-weighted turboRARE images (echo time TE = 36 ms, repetition time TR = 4200 ms, 2 averages) were first acquired from contiguous 1 mm thick axial slices for tumor localization and volume determination. Next, 2 sets of multigradient echo (MGE) images (TE = 6 to 28 ms, 4 ms echo spacing, TR = 200 ms, flip angle α = 45°, 8 averages, acquisition time AQ = 3 min 37 s) were acquired from 3 axial 1 mm slices through the tumor center while the mouse breathed air. The gas supply was then switched to carbogen, and following a 10-minute transition period, a further identical MGE image set was acquired. The gas supply was then reverted back to air and, after another 10-minute transition period, another MGE image set was acquired. A final MGE image set was then acquired 1 minute after intravenous injection of 150 μmol/kg USPIO particles (ferumoxtran-10, Sinerem; Guerbet, Villepinte, France).

Tumor volumes were determined using segmentation from regions of interest (ROIs) drawn on T_2_-weighted images for each tumor-containing slice, using in-house software (Imageview, developed in IDL; ITT Visual Information Systems, Boulder, CO). MGE data were analyzed using a Bayesian maximum a posteriori approach.^20^ This modeled the MGE signal magnitude as a single exponential decay and took into account its Rician distribution. Furthermore, it enabled estimates of ΔR_2_^∗^ uncertainty to be defined and the probability that a given ΔR_2_^∗^ estimate was significantly greater than or less than zero. Thus, the number of voxels within the tumor ROI with an uncertainty of less than 0.05 ms^−1^ and with a significant (*P* < 0.05) change in R_2_^∗^ induced by carbogen (ΔR_2_^∗^_carbogen_) and/or USPIO particles (ΔR_2_^∗^_USPIO_) were calculated. Fractional blood volume (ξ, %) was quantified from the ΔR_2_^∗^_USPIO_ data as previously described.^21^ The increase in blood susceptibility caused by the USPIO particles was extrapolated from a previously published value for a dose of 200 μmol/kg.^22^

RGB maps were then generated, with the red channel designated to voxels with a positive ΔR_2_^∗^_carbogen_, the blue channel to voxels with negative ΔR_2_^∗^_carbogen_, and the green channel to positive ΔR_2_^∗^_USPIO_. It was assumed that ΔR_2_^∗^_USPIO_ could only be positive, and negative values were assumed to be a result of uncertainty in R_2_^∗^ estimates. Regions with both negative ΔR_2_^∗^_carbogen_ and positive ΔR_2_^∗^_USPIO_ therefore appeared cyan (blue + green), and regions with both positive ΔR_2_^∗^_carbogen_ and positive ΔR_2_^∗^_USPIO_ appeared yellow (red + green).

### Histological Assessment of Tumor Perfusion

Following MRI, Hoechst 33342 (15 mg/kg in water; Sigma, Poole, UK) was administered intravenously via a lateral tail vein. Hoechst 33342 is a nuclear dye that stains the cells lining blood vessels that are perfused at the time of injection, affording a measure of functional tumor vasculature.^23^ Mice were killed 1 minute after injection of Hoechst 33342 and tumors rapidly excised and snap frozen over liquid nitrogen. Contiguous 10 μm frozen sections at three levels through each tumor were cut on a cryotome in approximately the same plane as was imaged with MRI. Sections were fixed in ice-cold acetone for 10 minutes before being mounted in phosphate-buffered saline. Hoechst 33342 fluorescence signals from whole tumor sections were then recorded at 365 nm using a motorized scanning stage (Prior Scientific Instruments, Cambridge, UK) attached to a BX51 microscope (Olympus Optical, London, UK) driven by CellP (Soft Imaging System, Munster, Germany). The area of Hoechst 33342 fluorescence as a percentage of the total tumor area mean Hoechst perfused area (mHPA)] was then calculated using CellP.

### Statistical Analysis

All data are reported as mean of median values ± 1 standard error of the mean unless otherwise stated. Statistical significance was determined using Student's 2-tailed unpaired *t* test assuming 2 samples of the population with equal variance. A *P* value of < 0.05 was considered significant.

## RESULTS

### MRI-Derived Tumor Volumes

The small tumor cohort of subcutaneous PC3 LN3 xenografts had a mean volume of 44 ± 9 mm^3^, which was significantly smaller than the large subcutaneous tumor cohort (563 ± 40 mm^3^, *P* < 0.01). The orthotopically propagated PC3 LN3 xenografts had a mean volume of 377 ± 134 mm^3^.

### Quantitation of Tumor R_2_^∗^, ΔR_2_^∗^_carbogen_, and Fractional Blood Volume

**FIGURE 1:**
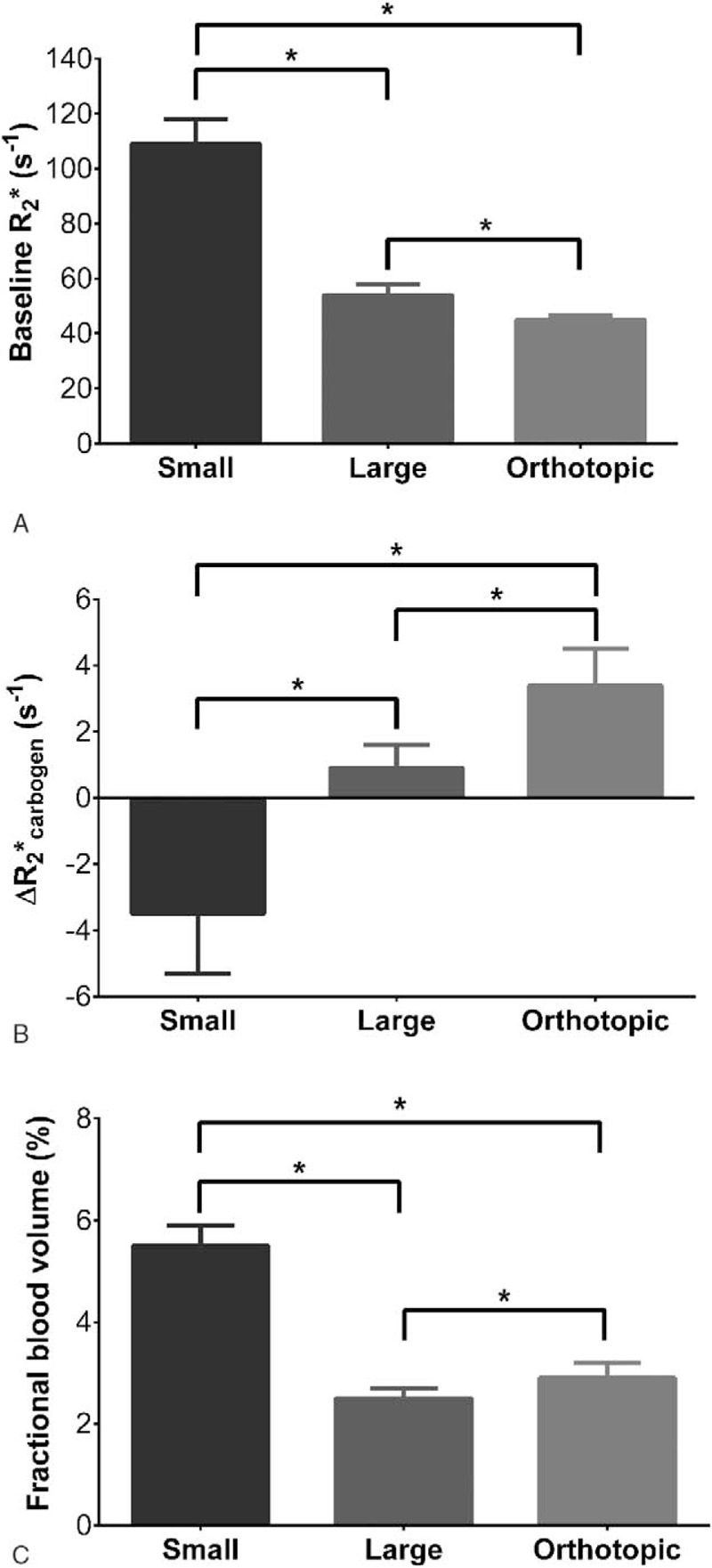
Summary of (A) baseline R_2_^∗^, (B) carbogen-induced ΔR_2_^∗^, and (C) fractional blood volume determined from the cohorts of small (n = 6) and large (n = 6) subcutaneous, and orthotopically propagated PC3 LN3 prostate cancer xenografts (n = 5). Values are mean ± 1 s.e.m, ^∗^*P* < 0.05, Student's 2-tailed unpaired *t* test.

Small and large subcutaneous, and orthotopic PC3 LN3 tumors exhibited significantly different mean baseline R_2_^∗^, and ΔR_2_^∗^_carbogen_ (Fig. 1A, B). The mean baseline R_2_^∗^ was significantly faster in the small subcutaneous tumor cohort than in the large subcutaneous or orthotopic PC3 LN3 tumors, and orthotopic tumors exhibited a significantly slower baseline R_2_^∗^ than the large subcutaneous tumors. Mean ΔR_2_^∗^_carbogen_ was negative in the small subcutaneous tumor cohort, and positive in both the large subcutaneous and orthotopic cohorts. The mean fractional tumor blood volume was significantly greater in the small subcutaneous tumors than the large subcutaneous and orthotopic tumors (Fig. 1C). Fractional blood volume was significantly higher in the orthotopic cohort, than the large subcutaneous group.

### Combined Carbogen-Ultrasmall Superparamagnetic Iron Oxide Imaging Response Categories

**FIGURE 2:**
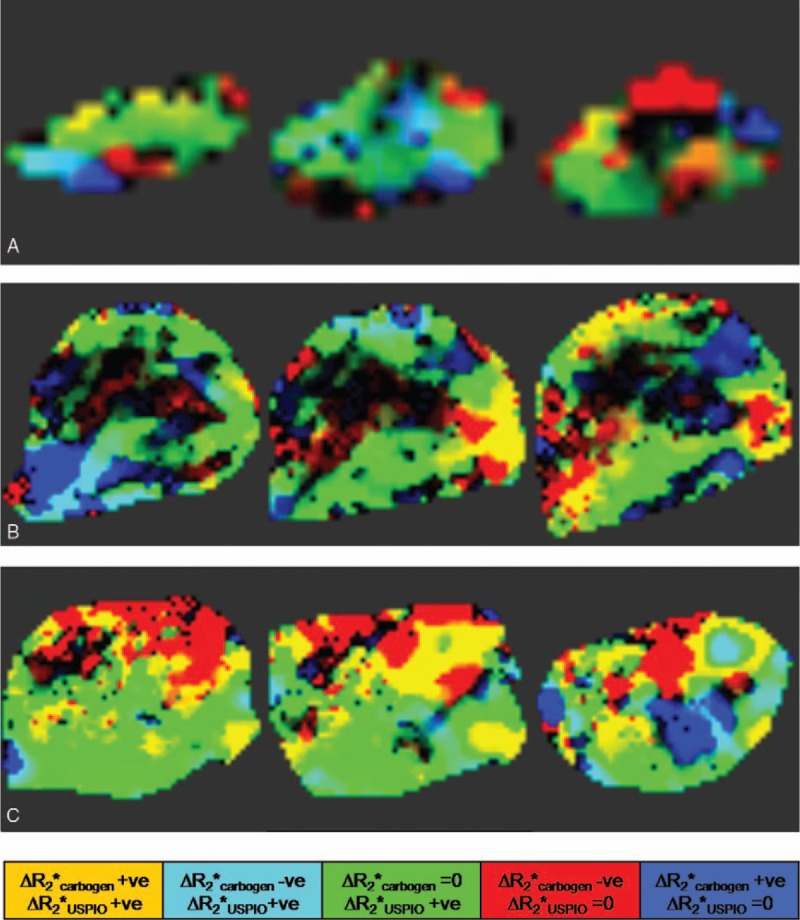
CUSPIO RGB maps from the three axial slices obtained from representative (A) small subcutaneous, (B) large subcutaneous, and (C) orthotopic PC3 LN3 prostate xenografts. The key shows the color-coded CUSPIO response categories.

Representative CUSPIO RGB maps, which show the spatial distribution of R_2_^∗^ responses to carbogen breathing and USPIO particle injection, for small and large subcutaneous, and orthotopic PC3 LN3 tumors are shown in Fig. 2. Visual inspection of the RGB maps from all tumor cohorts showed a heterogeneous spatial distribution of the 5 CUSPIO response categories. The RGB maps from each of the PC3 LN3 tumor cohorts revealed a differing spatial distribution of CUSPIO response categories. The large subcutaneous tumors typically showed a central nonresponding region, which were less prevalent in the small or orthotopic tumors. Orthotopic tumors appeared to have larger continuous regions of green and cyan voxels contained in one area of the tumor, whereas the subcutaneous tumors exhibited several smaller, more discrete regions of green voxels distributed across the tumor. A greater incidence of yellow voxels was noticeable in the orthotopic tumor RGB maps, than the subcutaneous tumor cohorts, which agrees with the quantified CUSPIO data.

The predicted predominant response of tumor tissue to carbogen breathing and USPIO particles is a negative ΔR_2_^∗^_carbogen_ followed by positive ΔR_2_^∗^_USPIO_, represented by cyan voxels on the RGB maps. It was therefore interesting that the RGB maps for all the PC3 LN3 tumors showed regions where there was significant ΔR_2_^∗^_carbogen_ but no significant ΔR_2_^∗^_USPIO_ (red and blue voxels), and regions where there was no significant ΔR_2_^∗^_carbogen_ followed by a significant ΔR_2_^∗^_USPIO_ (green voxels). The CUSPIO response categories in PC3 LN3 prostate tumors were separated spatially into regions that were larger than a single voxel (0.23 × 0.23 mm^2^), but small compared with the entire tumor ROI.^2^

To quantify the spatial information displayed in the CUSPIO RGB maps, the percentage of tumor ROI voxels that exhibited each R_2_^∗^ response category was calculated, and is summarized in Table 1. No significant difference in any of the 5 CUSPIO response categories was measured between the small and large subcutaneous PC3 LN3 tumor cohorts. A significantly greater percentage of the tumor ROI was occupied by red and yellow voxels in the orthotopic cohort, than the small subcutaneous tumors. A significantly smaller number of blue voxels was measured in the orthotopic cohort, than the small subcutaneous tumors.

### Hoechst 33342 Fluorescence Microscopy

**FIGURE 3:**
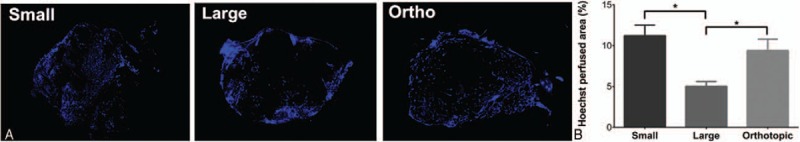
A, Composite fluorescence microscopy images showing Hoechst 33342 uptake in representative small and large subcutaneous, and orthotopic PC3 LN3 prostate xenografts. B, Summary of Hoechst 33342 perfused area (HPA%), which represents plasma perfusion, measured by fluorescence microscopy in small (n = 6) and large (n = 6) subcutaneous, and orthotopic (n = 5) PC3 LN3 prostate tumors. Values are mean ± 1 s.e.m., ^∗^*P* < 0.05, Student's 2-tailed unpaired *t* test.

Composite fluorescence images of Hoechst 33342 uptake from small and large subcutaneous tumors and an orthotopic PC3 LN3 xenograft are shown in Fig. 3A. Hoechst 33342 uptake was heterogeneous in all three tumor cohorts, and visually greater in the center of the small subcutaneous and orthotopic tumors. Significantly greater mHPA was determined in both the small subcutaneous and orthotopic cohorts than the large subcutaneous tumor group (Fig. 3B).

## DISCUSSION

In this study, the CUSPIO imaging protocol was used to investigate how tumor size and site of implantation affect the vascular characteristics and function of PC3 LN3 prostate tumors *in vivo*. PC3 LN3 prostate tumors were propagated subcutaneously and orthotopically, and the subcutaneous tumors were separated into small and large tumor cohorts. All tumors were then imaged using the CUSPIO imaging protocol. It was hypothesized that tumor size and site of implantation would affect the resulting vascular phenotype.

### Tumor Baseline R_2_^∗^, ΔR_2_^∗^_carbogen_, and Fractional Blood Volume

As the oxygenation of hemoglobin is proportional to the arterial blood p_a_O_2_, and therefore in equilibrium with tissue pO_2_, measurements of baseline R_2_^∗^ have been exploited as a sensitive biomarker of hypoxia.^11,24^ The significantly faster baseline R_2_^∗^ determined in the small subcutaneous PC3 LN3 xenografts suggests that tumors from this cohort were the most hypoxic. In order to progress, such small tumors are likely to initiate a strong hypoxia-driven vasculogenic drive, with associated increase expression of vascular endothelial growth factor (VEGF), resulting in immature, unstable vasculature transporting predominantly deoxygenated blood, resulting in a relatively faster R_2_^∗^. Magnetic field inhomogeneties arising from susceptibility artifacts caused by air-tissue interfaces, which may be more prevalent when imaging such small tumors, would also impact on the R_2_^∗^ measurement, though the use of dental paste to immobilize the tumors should limit this.^19^

Tumor ΔR_2_^∗^_carbogen_ was significantly different across all three tumor cohorts, suggesting that both the size and site of implantation of the PC3 LN3 tumors had an effect on their incipient hemodynamic vasculature.^25^ Differences in functional vasculature between subcutaneous and orthotopic tumors reflect a difference in the interaction of tumor vessels with the host vasculature, or differential pericyte/endothelial cell recruitment by different host tissues.^26,27^ Tumor-stromal interactions are powerful determinants of vascular growth factor expression, and therefore the resulting vasculature.^26,28^ Furthermore, growth factor receptor expression differs in endothelial cells from different tissues, which may be reflected in these differences in MRI vascular biomarkers.^29^ Differences between small and large subcutaneous xenografts is likely a consequence of more mature vasculature, higher interstitial pressure, and increasing lack of vascular hierarchy associated with larger tumours.^30^ The small subcutaneous tumor cohort exhibited a negative ΔR_2_^∗^_carbogen_, indicative of a functional, erythrocyte perfused vascular network.^11^ Combined with their significantly faster baseline R_2_^∗^, these data suggest that the small PC3 LN3 tumors were hypoxic at baseline, and become less hypoxic during carbogen breathing.^31^ The positive ΔR_2_^∗^_carbogen_ measured in the large subcutaneous and orthotopic tumors can be explained by the vascular steal effect, that is, the distribution of blood away from the tumor vascular bed by the systemic vasculature.^32,33^ From this, it can be inferred that both these cohorts have less hemodynamic vasculature than the small subcutaneous tumors.

The significantly greater fractional blood volume in the small subcutaneous PC3 LN3 tumors is consistent with previous work showing that small tumors are often better perfused.^30^ Vasculogenesis in small tumors provides a nutritive blood supply, but which may not meet the evolving metabolic adaptation/demands of the cancer cells, hence their markedly elevated hypoxia. As tumors progress, their rapid growth outpaces angiogenesis, resulting in a more poorly organized vascular hierarchy that, in conjunction with raised interstitial fluid pressure, causes plasma perfusion as a percentage of tumor volume to decrease.^27^ Hoechst 33342 uptake showed close agreement with the MRI-derived fractional blood volume results from the same subcutaneous tumors, providing further validation of this imaging biomarker.^34,35^ Orthotopic PC3 LN3 tumors exhibited a significantly larger blood volume than the large subcutaneous tumors, reflecting differences in vascular phenotype associated with the different tumor microenvironments, as there was no significant difference in the mean volumes of these tumor cohorts. This is consistent with the elevated vascular volume, measured by MRI, in orthotopic, compared with subcutaneous, PC3 tumors.^36^ These differences may arise from tumor cell interactions with stromal cells from the prostate compared with dermal site, or because of differential angiogenic growth factor receptor expression in the different tissues.

### Combined Carbogen-Ultrasmall Superparamagnetic Iron Oxide Imaging

The CUSPIO RGB maps from all 3 PC3 LN3 tumor cohorts revealed regions wherein R_2_^∗^ changed significantly (+ or -) during carbogen breathing, but did not change after administration of USPIO particles (blue and red voxels), as previously observed in other tumor models.^2^ This suggests that these regions have experienced vascular shutdown in the 15-minute period between carbogen inhalation and injection of USPIO particles. RGB maps from all tumors also showed a heterogeneous spatial distribution of ΔR_2_^∗^_USPIO_ (green) voxels, which is indicative of heterogeneous plasma perfusion. Green CUSPIO voxels have been previously shown to have a close spatial association with Hoechst 33342 uptake.^2^ RGB maps from the orthotopic PC3 LN3 tumors showed localized regions of green and cyan voxels, compared with more heterogeneous distribution seen in the subcutaneous tumors. This may reflect a difference in vascular supply to the tumors, resulting from the different microenvironments.^9^

There was no significant difference in any of the CUSPIO ΔR_2_^∗^ response categories between the small and large subcutaneous tumors, suggesting a similar incidence of transient hypoxia and levels of plasma perfusion in these 2 cohorts. Tumor size may therefore only weakly influence the incidence of intermittent blood flow and plasma perfusion, compared with tumor-stroma interactions and the effect of site of implantation. The similarity in the yellow and cyan CUSPIO response categories in the small and large tumors suggests that these two models had similarly patent, functional vasculature.

Quantitation of the CUSPIO categories revealed that orthotopic PC3 LN3 tumors exhibited a significantly greater percentage of red and yellow voxels, and a significantly smaller number of blue voxels, than the small subcutaneous cohort. The lower number of blue voxels suggests a lower incidence of transient hypoxia in the orthotopic tumors. The greater number of yellow voxels also suggests that the orthotopic tumors experienced a lower incidence of intermittent blood flow, as more of the tumor remained perfused throughout the whole CUSPIO imaging session than the small subcutaneous tumors. The greater percentage of red voxels, combined with the higher incidence of yellow voxels, in the orthotopic tumors, may indicate a greater incidence of vascular steal. This inference aligns with the positive mean ΔR_2_^∗^_carbogen_ measured in the orthotopic tumors, compared with the negative mean ΔR_2_^∗^_carbogen_ measured in the small subcutaneous cohort. Numerous factors are important determinants of the vascular phenotype that develops in tumors, including VEGF expression, endothelial cell VEGF receptor expression, and platelet-derived growth factor receptor (PDGFR-β) expression, and are all influenced by tumor-stroma interactions. The differences in CUSPIO response categories measured between small subcutaneous and orthotopic PC3 LN3 tumors may therefore reflect differences in pericyte coverage and vascular density in these two models, and which may also be influenced by the vascular bed at each anatomical site.^26,28,37,38^

Efficient tumor plasma perfusion is a key determinant of drug delivery, so a clear understanding on the extent, patency, and spatial distribution of functional tumor vasculature *in vivo* provided by techniques such as CUSPIO imaging is imperative for appropriate tumor model selection when designing pre-clinical therapy trials. Carbogen inhalation has been used both clinically and pre-clinically to increase drug uptake in solid tumors through hypercapnia-induced vasodilation.^39–43^ The potential to use CUSPIO imaging to noninvasively assess the degree and heterogeneity of vascular functionality, tumor oxygenation, and vascular shutdown will impact on both chemotherapy and radiotherapy, which generally exhibit higher efficacy in well oxygenated tumor tissue.^44^ Differences in vascular architecture and function arising in subcutaneous and orthotopic models also has implications for the sensitivity of a model in which to evaluate novel anti-angiogenic drugs, and targeted agents whose mode of action is predicted to elicit an anti-angiogenic effect.^3^

In conclusion, the vascular structure and function of tumor xenografts, derived from a PC3 LN3 human prostate cell line, was shown to be dependent on both the site of implantation and relative tumor size using the CUSPIO imaging protocol. Significant differences in ΔR_2_^∗^_carbogen_, fractional blood volume, and CUSPIO response categories were determined between small and large subcutaneous, and orthotopically propagated PC3 LN3 prostate cancer xenografts.

## Figures and Tables

**TABLE 1:** CUSPIO Mean Fractional Response Categories for Small and Large Subcutaneous, and Orthotopic PC3 LN3 Prostate Tumors

	ΔR_2_^*^_carbogen_ +ve	ΔR_2_^*^_carbogen_ −ve	ΔR_2_^*^_carbogen_ = 0	ΔR_2_^*^_carbogen_ −ve	ΔR_2_^*^_carbogen_ +ve
	ΔR_2_^*^_USPIO_ +ve	ΔR_2_^*^_USPIO_+ve	ΔR_2_^*^_USPIO_ +ve	ΔR_2_^*^_USPIO_ = 0	ΔR_2_^*^_USPIO_ = 0
Small	10.1 ± 5	14.3 ± 4	22.2 ± 6	10.9 ± 5	17.5 ± 5
Large	11.1 ± 1	9.0 ± 2	25.8 ± 4	14.0 ± 3	9.6 ± 2
Orthotopic	26.8 ± 5^*^	11.0 ± 2	22.5 ± 3	17.7 ± 3^*^	5.9 ± 2^*^

Values are mean % of the total tumor ROI ± 1 s.e.m.

^*^P < 0.05, Student's 2-tailed unpaired *t* test, between small and orthotopic tumors.

## References

[1] Jung CW Jacobs P Physical and chemical properties of superparamagnetic iron oxide MR contrast agents: ferumoxides, ferumoxtran, ferumoxsil. *Magn Reson Imaging* 1995; 13:661–674. 8569441 10.1016/0730-725x(95)00024-b

[2] Burrell JS Walker-Samuel S Baker LC Investigating temporal fluctuations in tumor vasculature with combined carbogen and ultrasmall superparamagnetic iron oxide particle (CUSPIO) imaging. *Magn Reson Med* 2011; 66:227–234. 21305600 10.1002/mrm.22779

[3] Burrell JS Walker-Samuel S Baker LCJ Evaluation of novel combined carbogen USPIO (CUSPIO) imaging biomarkers in assessing the antiangiogenic effects of cediranib (AZD2171) in rat C6 gliomas. *Int J Cancer* 2012; 131:1854–1862. 22290271 10.1002/ijc.27460

[4] Ellis LM Fidler IJ Finding the tumor copycat. Therapy fails, patients don’t. *Nat Med* 2010; 16:974–975. 20823880 10.1038/nm0910-974

[5] Van Dyke T Finding the tumor copycat: approximating a human cancer. *Nat Med* 2010; 16:976–977. 20823881 10.1038/nm0910-976 PMC3533444

[6] de Bono JS Ashworth A Translating cancer research into targeted therapeutics. *Nature* 2010; 467:543–549. 20882008 10.1038/nature09339

[7] Workman P Aboagye EO Balkwill F Guidelines for the welfare and use of animals in cancer research. *Br J Cancer* 2010; 102:1555–1577. 20502460 10.1038/sj.bjc.6605642 PMC2883160

[8] Chesler L Weiss WA Genetically engineered murine models: contribution to our understanding of the genetics, molecular pathology and therapeutic targeting of neuroblastoma. *Semin Cancer Biol* 2011; 21:245–255. 21958944 10.1016/j.semcancer.2011.09.011 PMC3504935

[9] Walker-Samuel S Boult JK McPhail LD Non-invasive in vivo imaging of vessel calibre in orthotopic prostate tumour xenografts. *Int J Cancer* 2012; 130:1284–1293. 21469141 10.1002/ijc.26112

[10] Jamin Y Glass L Hallsworth A Intrinsic susceptibility MRI identifies tumors with *ALK*^*F1174L*^ mutation in genetically-engineered murine models of high-risk neuroblastoma. *PLoS One* 2014; 9:e92886. 24667968 10.1371/journal.pone.0092886 PMC3965493

[11] Robinson SP Rijken PF Howe FA Tumor vascular architecture and function evaluated by non-invasive susceptibility MRI methods and immunohistochemistry. *J Magn Reson Imaging* 2003; 17:445–454. 12655584 10.1002/jmri.10274

[12] Robinson SP Ludwig C Paulsson J The effects of tumor-derived platelet-derived growth factor on vascular morphology and function in vivo revealed by susceptibility MRI. *Int J Cancer* 2008; 122:1548–1556. 18033683 10.1002/ijc.23279

[13] Baker LCJ Boult JKR Walker-Samuel S The HIF-pathway inhibitor NSC-134754 induces metabolic changes and anti-tumour activity while maintaining vascular function. *Br J Cancer* 2012; 106:1638–1647. 22498643 10.1038/bjc.2012.131 PMC3349173

[14] Boult JKR Jamin Y Jacobs V False-negative MRI biomarkers of tumour response to targeted cancer therapeutics. *Br J Cancer* 2012; 106:1960–1966. 22596237 10.1038/bjc.2012.208 PMC3388570

[15] Ramasawmy R Campbell-Washburn AE Wells JA Hepatic arterial spin labelling MRI: an initial evaluation in mice. *NMR Biomed* 2015; 28:272–280. 25522098 10.1002/nbm.3251 PMC4670473

[16] Sanderson S Valenti M Gowan S Benzoquinone ansamycin heat shock protein 90 inhibitors modulate multiple functions required for tumor angiogenesis. *Mol Cancer Ther* 2006; 5:522–532. 16546966 10.1158/1535-7163.MCT-05-0439

[17] Workman P Aboagye EO Balkwill F Guidelines for the welfare and use of animals in cancer research. *Br J Cancer* 2010; 102:1555–1577. 20502460 10.1038/sj.bjc.6605642 PMC2883160

[18] Kilkenny C Browne WJ Cuthill IC Improving bioscience research reporting: the ARRIVE guidelines for reporting animal research. *PLoS Biol* 2010; 8:e1000412. 20613859 10.1371/journal.pbio.1000412 PMC2893951

[19] Landuyt W Sunaert S Farina D In vivo animal functional MRI: improved image quality with a body-adapted mold. *J Magn Reson Imaging* 2002; 16:224–227. 12203772 10.1002/jmri.10144

[20] Walker-Samuel S Orton M McPhail LD Bayesian estimation of changes in transverse relaxation rates. *Magn Reson Med* 2010; 64:914–921. 20806382 10.1002/mrm.22478

[21] Yablonskiy DA Haacke EM Theory of NMR signal behavior in magnetically inhomogeneous tissues: the static dephasing regime. *Magn Reson Med* 1994; 32:749–763. 7869897 10.1002/mrm.1910320610

[22] Tropres I Grimault S Vaeth A Vessel size imaging. *Magn Reson Med* 2001; 45:397–408. 11241696 10.1002/1522-2594(200103)45:3<397::aid-mrm1052>3.0.co;2-3

[23] Smith KA Hill SA Begg AC Validation of the fluorescent dye Hoechst 33342 as a vascular space marker in tumours. *Br J Cancer* 1988; 57:247–253. 3355762 10.1038/bjc.1988.54 PMC2246513

[24] McPhail LD Robinson SP Intrinsic susceptibility MR imaging of chemically induced rat mammary tumors: relationship to histologic assessment of hypoxia and fibrosis. *Radiology* 2010; 254:110–118. 20032145 10.1148/radiol.2541090395

[25] Robinson SP Rijken PF Howe FA Tumor vascular architecture and function evaluated by non-invasive susceptibility MRI methods and immunohistochemistry. *J Magn Reson Imaging* 2003; 17:445–454. 12655584 10.1002/jmri.10274

[26] Pietras K Ostman A Hallmarks of cancer: interactions with the tumor stroma. *Exp Cell Res* 2010; 316:1324–1331. 20211171 10.1016/j.yexcr.2010.02.045

[27] Gilead A Meir G Neeman M The role of angiogenesis, vascular maturation, regression and stroma infiltration in dormancy and growth of implanted MLS ovarian carcinoma spheroids. *Int J Cancer* 2004; 108:524–531. 14696116 10.1002/ijc.11583

[28] Fukumura D Xavier R Sugiura T Tumor induction of VEGF promoter activity in stromal cells. *Cell* 1998; 94:715–725. 9753319 10.1016/s0092-8674(00)81731-6

[29] Fidler IJ Ellis LM The implications of angiogenesis for the biology and therapy of cancer metastasis. *Cell* 1994; 79:185–188. 7525076 10.1016/0092-8674(94)90187-2

[30] Jain RK Baxter LT Mechanisms of heterogeneous distribution of monoclonal antibodies and other macromolecules in tumors: significance of elevated interstitial pressure. *Cancer Res* 1988; 48:7022–7032. 3191477

[31] Baker LCJ Boult JKR Jamin Y Evaluation and immunohistochemical qualification of carbogen-induced ΔR_2_^∗^ as a noninvasive imaging biomarker of improved tumor oxygenation. *Int J Radiat Oncol Biol Phys* 2013; 87:160–167. 23849692 10.1016/j.ijrobp.2013.04.051

[32] Karczmar GS River JN Li J Effects of hyperoxia on T_2_^∗^ and resonance frequency weighted magnetic resonance images of rodent tumours. *NMR Biomed* 1994; 7:3–11. 8068523 10.1002/nbm.1940070103

[33] Robinson SP Rodrigues LM Ojugo AS The response to carbogen breathing in experimental tumour models monitored by gradient-recalled echo magnetic resonance imaging. *Br J Cancer* 1997; 75:1000–1006. 9083335 10.1038/bjc.1997.172 PMC2222734

[34] Robinson SP Howe FA Griffiths JR Susceptibility contrast magnetic resonance imaging determination of fractional tumor blood volume: a noninvasive imaging biomarker of response to the vascular disrupting agent ZD6126. *Int J Radiat Oncol Biol Phys* 2007; 69:872–879. 17889267 10.1016/j.ijrobp.2007.06.061

[35] Walker-Samuel S Boult JKR McPhail LD Non-invasive in vivo imaging of vessel calibre in orthotopic prostate tumour xenografts. *Int J Cancer* 2012; 130:1284–1293. 21469141 10.1002/ijc.26112

[36] Penet M-F Pathak AP Raman V Noninvasive multiparametric imaging of metastasis-permissive microenvironments in a human prostate cancer xenograft. *Cancer Res* 2009; 69:8822–8829. 19861534 10.1158/0008-5472.CAN-09-1782 PMC2783669

[37] Pasqualini R Ruoslahti E Organ targeting in vivo using phage display peptide libraries. *Nature* 1996; 380:364–366. 8598934 10.1038/380364a0

[38] Aird WC Endothelial cell heterogeneity. *Crit Care Med* 2003; 31:S221–S230. 12682444 10.1097/01.CCM.0000057847.32590.C1

[39] McSheehy PM Port RE Rodrigues LM Investigations in vivo of the effects of carbogen breathing on 5-fluorouracil pharmacokinetics and physiology of solid rodent tumours. *Cancer Chem Pharm* 2005; 55:117–128. 10.1007/s00280-004-0851-9 15592719

[40] McSheehy PM Robinson SP Ojugo AS Carbogen breathing increases 5-fluorouracil uptake and cytotoxicity in hypoxic murine RIF-1 tumors: a magnetic resonance study in vivo. *Cancer Res* 1998; 58:1185–1194. 9515804

[41] Gupta N Saleem A Kotz B Carbogen and nicotinamide increase blood flow and 5-fluorouracil delivery but not 5-fluorouracil retention in colorectal cancer metastases in patients. *Clin Cancer Res* 2006; 12:3115–3123. 16707610 10.1158/1078-0432.CCR-05-0513

[42] Rodrigues LM Maxwell RJ McSheehy PM In vivo detection of ifosfamide by 31P-MRS in rat tumours: increased uptake and cytotoxicity induced by carbogen breathing in GH3 prolactinomas. *Br J Cancer* 1997; 75:62–68. 9000599 10.1038/bjc.1997.10 PMC2222708

[43] Rodrigues LM Robinson SP McSheehy PM Enhanced uptake of ifosfamide into GH3 prolactinomas with hypercapnic hyperoxic gases monitored in vivo by (31)P MRS. *Neoplasia* 2002; 4:539–543. 12407448 10.1038/sj.neo.7900259 PMC1503668

[44] Kirkpatrick JP Cardenas-Navia LI Dewhirst MW Predicting the effect of temporal variations in PO2 on tumor radiosensitivity. *Int J Radiat Oncol Biol Phys* 2004; 59:822–833. 15183486 10.1016/j.ijrobp.2004.02.015

